# Synthetic health data in Canada: A scoping review of methods, applications, and data sources

**DOI:** 10.17269/s41997-026-01153-6

**Published:** 2026-02-09

**Authors:** Hassan Maleki Golandouz, Lisa M. Lix

**Affiliations:** College of Community and Global Health, University of Manitoba, Winnipeg, MB Canada

**Keywords:** Data collection, Health information systems, Privacy, Artificial intelligence, Collecte de données, Systèmes d’information sur la santé, Vie privée, Intelligence artificielle

## Abstract

**Background:**

Access to provincial health-related data for multi-jurisdictional studies in Canada is restricted by privacy laws. Synthetic data (SD), which mimic real data, can facilitate privacy preservation. However, information on SD use in Canadian research is limited.

**Objectives:**

To review characteristics, methods, and applications of published studies generating SD from Canadian health data (HD), including administrative, survey, public health, and clinical sources.

**Methods:**

We conducted a scoping review following Arksey and O’Malley, Joanna Briggs Institute, and Preferred Reporting Items for Systematic reviews and Meta-Analyses extension for Scoping Reviews guidelines on studies (to September 2024) generating SD from provincial/national HD. We included English-language peer-reviewed articles and grey literature, identified through PubMed, Scopus, Web of Science, Google, and references. We extracted and descriptively analyzed data on HD types, research purposes, geographic sources, synthesis methods, and quality evaluation.

**Synthesis:**

Of 232 identified articles, 31 were reviewed and nine met inclusion criteria; three additional articles were found through references and Google. Eleven articles were peer-reviewed. Topics included data replication, bias mitigation, and privacy-risk assessment. Survey data were most commonly synthesized. SD were generated from national/provincial datasets, including Canadian Community Health Survey and administrative/clinical data from Alberta, Manitoba, British Columbia, and Ontario. Synthesis methods included generative, sampling, and predictive models. Data quality evaluations assessed replicability, privacy risk, and predictive performance.

**Conclusion:**

SD have mainly been used in single-province studies and national surveys. Broader use in clinical and public HD with methodological consistency could strengthen its role for privacy-protecting, multi-jurisdictional research and surveillance initiatives.

**Supplementary Information:**

The online version contains supplementary material available at https://doi.org/10.17269/s41997-026-01153-6.

## Résumé

**Contexte:**

Au Canada, l’accès aux données de santé provinciales pour mener des études multiterritoriales est limité par les lois sur le respect de la vie privée. Les données synthétiques (DS), qui imitent les données réelles, peuvent faciliter la protection de la confidentialité, mais les informations sur leur utilisation dans la recherche canadienne sont limitées.

**Objectifs:**

Nous avons cherché à examiner les caractéristiques, les méthodes et les applications d’études publiées ayant généré des DS à partir de données de santé canadiennes de sources administratives, cliniques, d’enquêtes et de santé publique.

**Méthodes:**

En suivant les lignes directrices d’Arksey et O’Malley, du Joanna Briggs Institute et de l’extension *PRISMA for Scoping Reviews* de PRISMA (Preferred Reporting Items for Systematic reviews and Meta-Analyses), nous avons mené une revue exploratoire des études ayant généré des DS à partir de données de santé provinciales/nationales (jusqu’en septembre 2024). Nous avons inclus les articles de langue anglaise évalués par les pairs et les articles de la littérature grise répertoriés dans PubMed, Scopus, Web of Science, Google et dans les références. Nous avons extrait et fait l’analyse descriptive des données en fonction des types de données de santé, des fins de recherche, des sources géographiques, des méthodes de synthèse et de l’évaluation de la qualité.

**Synthèse:**

Sur les 232 articles recensés, nous en avons examiné 31; neuf respectaient les critères d’inclusion; trois autres articles ont été trouvés dans des références et par Google. Onze articles étaient évalués par les pairs. Les sujets abordés étaient la réplication de données, l’atténuation des biais et l’évaluation des risques pour la vie privée. Les données le plus souvent synthétisées étaient les données d’enquêtes. Les DS étaient générées à partir d’ensembles de données nationaux/provinciaux, dont l’Enquête sur la santé dans les collectivités canadiennes et les données administratives/cliniques de l’Alberta, du Manitoba, de la Colombie-Britannique et de l’Ontario. Les méthodes de synthèse incluaient des modèles génératifs, d’échantillonnage et de prévision. Les évaluations de la qualité des données portaient sur la réplicabilité, le risque pour la vie privée et la performance prédictive.

**Conclusion:**

Les DS sont principalement utilisées dans des études monoprovinciales et des enquêtes nationales. L’élargissement de leur utilisation aux données de santé clinique et de santé publique, en assurant la cohérence méthodologique, pourrait renforcer leur rôle dans les initiatives de protection de la vie privée, de recherche multiterritoriale et de surveillance.

## Introduction

Accessing provincial/territorial health-related data for multi-jurisdictional studies in Canada is difficult due to privacy laws that prevent sharing individual-level data across provincial/territorial borders. Factors limiting data accessibility is concern over patient privacy and regulatory frameworks governing data sharing across jurisdictions. Privacy considerations often challenge the accessibility of health data (Kalkman et al., 2019). Canada's decentralized healthcare system poses additional challenges for researchers aiming to conduct multi-jurisdictional studies. Although universal healthcare coverage facilitates population-wide research by enabling data harmonization, integration, and linkage (Council of Canadian Academies: Expert Panel on Timely Access to Health and Social Data for Health Research and Health System Innovation, 2015), the fragmentation of healthcare administration across provincial and territorial jurisdictions complicates such efforts.

Provinces and territories create many of their own health databases with distinct structures and governance policies, making it difficult to combine person-specific data for multi-jurisdictional studies. While certain datasets—such as prescription data (Canadian Institute for Health Information, 2025)—may be partially harmonized or shared through national reporting systems, significant differences in privacy rules, ethics review processes, and legal frameworks often prevent full data integration. As a result, researchers commonly rely on distributed methods (McGrail et al., 2020), where analyses are conducted separately within each jurisdiction and only aggregate results are shared. This approach requires expertise in managing large datasets and understanding the specific data collection and governance practices unique to each jurisdiction (Lavoie et al., 2016). However, inconsistencies in governance, lengthy approval processes, and high costs often make data access burdensome, discouraging researchers from conducting cross-jurisdictional studies (Katz et al., 2018). As a result, despite Canada’s rich health-related data resources, the complexity of accessing and using them limits their potential. The urgency of efficient data sharing became especially clear during the COVID-19 pandemic, when delays in accessing health data hampered rapid research and public health responses (Downey, 2020; Fazlioglu, 2021; Layne et al., 2020).

A promising approach to facilitate access to health-related data for research while maintaining privacy and ethical standards, is synthetic data (SD) generation. SD refers to artificially created datasets that retain the statistical properties of real-world health data while eliminating direct identifiers, thereby reducing privacy risks (El Emam et al., 2020 El Emam & Hoptroff, 2019). Several studies have highlighted that SD poses minimal identity disclosure risk compared to traditional de-identified datasets (Hu et al., 2014; Reiter, 2004, 2005; Ruiz et al., 2018; Taub et al., 2018; Wei & Reiter, 2016), and empirical evaluations have further supported the notion that SD carries a low risk of re-identification (El Emam et al., 2020).

The generation of SD presents an opportunity to overcome some barriers associated with traditional data-sharing models. By creating realistic yet non-identifiable versions of sensitive datasets, SD allows researchers to conduct meaningful analyses without directly handling identifiable patient information. This approach has the potential to unlock historically siloed health data, enabling broader access for secondary research purposes.

Synthetic datasets have been developed and made publicly available for health research internationally. Examples include the National COVID Cohort Collaborative (N3C) dataset maintained by the National Institutes of Health (NIH; Haendel et al., 2021), the Centers for Medicare & Medicaid Services (CMS) Data Entrepreneur’s Synthetic Public Use Files (Centers for Medicare & Medicaid Services (CMS),n.d.), and synthetic cardiovascular and COVID-19 datasets from the Clinical Practice Research Datalink (CPRD) in the United Kingdom (Clinical Practice Research Datalink (CPRD), n.d.; Wang et al., 2019). Other publicly accessible datasets include Accident and Emergency (A&E) data from the National Health Service (NHS) England (National Health Service (NHS) England, n.d.), cancer datasets from Public Health England (Simulacrum, n.d.), and a synthetic registry from the Dutch cancer registry (Integraal Kankercentrum Nederland (IKNL), n.d.). Additionally, synthetic versions of the French public health system claims and hospital datasets (SNDS) (Système National des Données de Santé, n.d.) and datasets derived from South Korea’s Health Insurance Review and Assessment Service (Ministry of Health and Welfare & Health Insurance Review & Assessment Service (HIRA), 2020) have been made available. Furthermore, a growing number of researchers are generating and publicly sharing synthetic versions of datasets used in their studies to promote open science (Lun et al., 2024).

While synthetic health data has gained traction internationally, development and use of these data within the Canadian context has not been investigated. The objective of this review was to examine the characteristics, methods, and outcomes of studies that generate synthetic health data that are based on real-world Canadian health data. By synthesizing existing evidence, this review aimed to evaluate the potential role of SD in facilitating health research in Canada while addressing current barriers to data access and privacy concerns.

## Methods

### Study design and framework

We conducted a scoping review following the methodological framework proposed by Arksey and O’Malley (Arksey & O’Malley, 2005), Joanna Briggs Institute (JBI; Peters et al., 2020), and Preferred Reporting Items for Systematic reviews and Meta-Analyses extension for Scoping Reviews (PRISMA-ScR; Tricco et al., 2018) guidelines to map the existing literature on the generation and use of synthetic health data derived from real Canadian health data.

### Eligibility criteria

Articles were included if they focused on the generation and use of synthetic health data derived from real Canadian health data, including administrative, survey, public health and clinical datasets. Eligible sources included English-language peer-reviewed research articles and grey literature identified through a structured Google search, with no date limits or critical appraisal. To streamline and facilitate the search, it was limited to articles with at least one author affiliated with a Canadian institution. An article qualified if it explicitly used real-world Canadian health data as a source for SD generation, even if additional datasets were involved.

### Information sources and search strategy

To identify relevant articles, we searched PubMed, Scopus, and Web of Science databases, and also relied on the Google Search engine. All databases were comprehensively searched up to September 2024, and an updated conducted in October 2024. These databases were chosen for their comprehensive coverage of published literature. We also examined the reference lists of the included articles to aid in identifying additional relevant studies. The search strategy combined keywords and Boolean operators related to SD generation, different types of health data, and Canadian affiliations. To increase the likelihood of capturing studies that used Canadian data, we limited our search to publications where at least one author was affiliated with a Canadian university. Key search terms included variations of "synthetic data," "data synthesis," "administrative health data," "clinical data," and "survey health data". The detailed search expressions are provided in Online Resource 1.

### Article selection

All retrieved records were imported into Rayyan, a literature review tool (Ouzzani et al., 2016). Duplicates were removed. One of the authors screened the titles and abstracts against the eligibility criteria. The full text was reviewed for all articles that appeared potentially relevant to the scoping review. The second author was consulted where the reviewer was uncertain about inclusion.

### Data extraction process and analysis

Data were extracted using a standardized data extraction form developed through a review of prior studies on synthetic health data generation (Mosquera et al., 2023; Pezoulas et al., 2024). Article characteristics included journal name and publication year. We recorded the type of health data used (e.g., administrative, survey, public health, clinical) and their structure (e.g., cross-sectional, longitudinal) used to generate SD. We also noted the geographic source (e.g., province/territory) and the time frame during which the health data were collected. The number of individuals or records in the real dataset was recorded. We identified the synthesis method used in the studies, such as statistical imputation (Cook et al., 2024) or generative adversarial networks (Juwara et al., 2024). Where applicable, we also noted synthesis model hyperparameters, adjustable settings in machine learning algorithms that influence how models are trained; examples include the learning rate, batch size, and number of training epochs. We also identified the software packages or platforms used for data synthesis, such as Synthpop (Nowok et al., 2016) in R and SDV (Patki et al., 2016) in Python. We recorded approaches used in the studies to evaluate the quality of the synthesized data, such as statistical similarity metrics (Sallier, 2020), predictive model performance (Cook et al., 2024), and disclosure risk assessments (El Emam et al., 2022). Study data were descriptively analyzed using frequencies and percentages. Narrative techniques were also used to synthesize article features, such as the purpose and findings/outcomes.

## Results

### Article selection

**Fig. 1:**
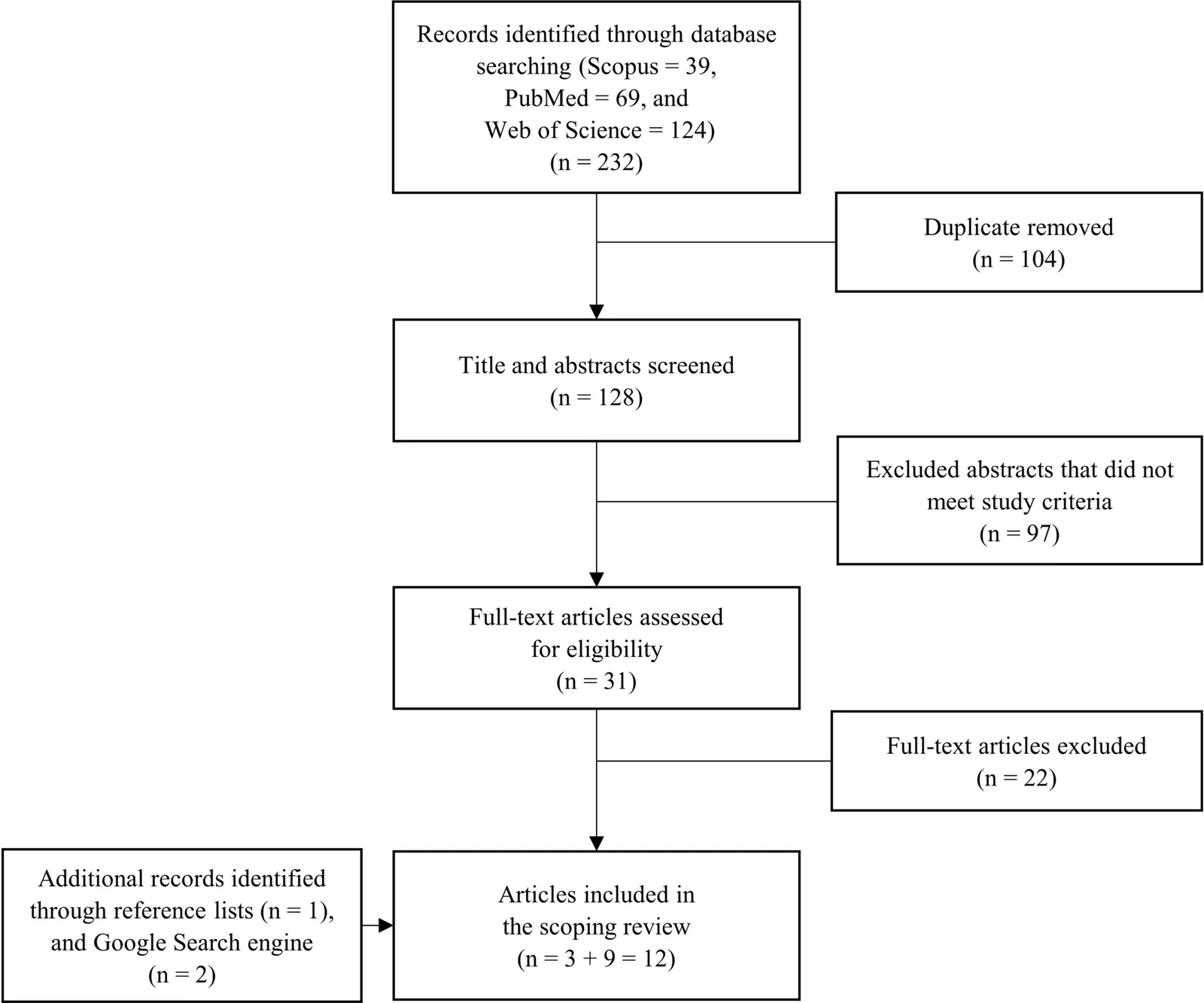
Flow chart of included and excluded articles

The search strategy yielded a total of 232 articles. After removing duplicates and after screening the title and abstracts based on the inclusion criteria, 31 articles were selected for full-text review. From these 31, nine articles met the inclusion criteria. We found one additional article through a review of article reference lists and two additional articles were identified using the Google Search engine. One of these was grey literature, and the other was not indexed in the databases searched. In the end, twelve articles were selected for data extraction (see Fig. 1).

### Characteristics of included articles

The articles were published between 2015 and 2024, with a notable increase in publication activity in 2023 and 2024 (Table 1). In fact, 8 of the 13 articles appeared during these two years. *Scientific Reports* and *JAMIA Open* each published two articles. Of the remaining articles, one appeared in a Canadian journal (*Journal of Medical Internet Research*), five in various international journals (e.g., *BMC Medical Research Methodology*, *International Journal of Population Data Science*), and one in a conference proceeding (*IEEE International Instrumentation and Measurement Technology Conference*). The articles focused on various aspects of SD use, ranging from evaluating the replicability of results and mitigating bias to validating privacy risks and using SD for educational and training purposes.

**Table 1:** Summary of articles included in the scoping review

Authors, date	Study purpose	Data type	Real-world data source	Real data sample size	Data structure	Synthetic data generation method and type	Programing language or software packages	Utility assessment	Privacy assessment	Reporting optimal model hyperparameters or the availability of source code (Yes/No/Available upon Request)
Cook et al., 2024	To improve brain age prediction models using synthetic EEG data augmentation	Clinical	EEG dataset from clinical records from over six years across seven hospitals in British Columbia, Canada	7,001	T	CGAN (G), WGAN (G), and WGAN-GP (G)	NR	Kolmogorov–Smirnov test for synthetic data realism, and using MAE and RMSE to assess predictive performance of SVR, LSTM and RFR models with varying levels of synthetic data integration	NR	No
El Emam et al., 2024	Examining the ability of synthetic data to replicate results from real data	Survey	Canadian Community Health Survey, 2014	903	C	Sequential decision trees (P), CTGAN (G)	R and Python	Eight replicability metrics: decision agreement, estimate agreement, standardized difference, confidence interval overlap, bias, confidence interval coverage, statistical power, precision (empirical standard error)	Membership disclosure metric	Yes
Juwara et al., 2024	Mitigating covariate bias in health data	Survey	Canadian Community Health Survey, 2014	63,522	C	Sequential decision trees (P)	R	AUROC, calibration (Brier score), precision of parameter estimates, confidence interval overlap, and fairness metrics	NR	Yes
Mosquera et al., 2023	Generating synthetic administrative health data	Administrative	Alberta administrative health data	120,000	L	LSTM (G)	Python	Hellinger distance, Cox regression hazard ratios for mean confidence interval overlap	Attribution disclosure risk	Available upon Request
Ayilara et al., 2023	Generating synthetic administrative health data	Administrative	Manitoba administrative health data	1,604,734	L	OSIM2 (S), ModOSIM (S)	SAS	Estimating the agreement of prescription drug use measures in PRDR, OSIM2, and ModOSIM using the concordance coefficient, and describing the synthetic and real data using frequencies and percentages	NR	No
Azizi et al., 2023	To compare synthetic data generation and federated analysis for international comparative studies	Survey	Canadian Community Health Survey, 2014	63,522	C	Sequential decision trees (P)	NR	Consistency of regression results	Membership disclosure test	No
El Emam et al., 2022	Validating privacy risk metrics	Public health, survey	Ontario COVID-19 Case Dataset and the Canadian Community Health Survey data	For each dataset, subsets of 5 k, 15 k, and 25 k samples were used	C	Sequential decision trees (P), CTGAN (G)	NR	NR	Membership disclosure F1 score	No
El Emam et al., 2021	Evaluate the utility of synthetic data by comparing analysis results between real and synthetic data	Public health, administrative	Ontario’s COVID-19 Case Dataset linked with community comorbidity, demographic, and socioeconomic characteristics	90,514 samples from linked datasets	C	Gradient boosted classification tree (P)	NR	AUROC, AUPRC, comparison of key predictors, confidence interval overlap	Attribute disclosure risks, membership disclosure risks	No
CanPath, 2021	Provide hands-on data analysis experience for students and faculty, raise awareness, and protect privacy	Survey	Canadian Partnership for Tomorrow (CanPath)	NR	C	NR	R Package ‘synthpop’	NR	NR	No
El Emam et al., 2020a	Developing and applying a methodology for evaluating the identity disclosure risks of fully synthetic data	Public health	Canadian COVID-19 Case Database	20,045	C	Sequential decision tree (P)	NR	NR	Identity disclosure risk model, meaningful identity disclosure risk	No
Sallier, 2020	To deliver synthetic data with high analytical value for users while protecting confidentiality	Administrative	Canadian Mortality Registry, Canadian Cancer Registry	NR	C	Multiple Imputation (P), FCS (P), CART (P)	R package ‘synthpop’	Comparison of marginal distributions, descriptive statistics of continuous variables, cross-tabulations, logistic regressions, survival analyses (Cox proportional hazard model)	Confidentiality evaluation with matching-based criteria	No
Hennessy et al., 2015	To develop a synthetic database for use in microsimulation models to investigate alternative health care financing strategies in Canada	Survey, administrative	Canadian Community Health Survey, Canadian Health Measures Survey, Survey of Household Spending, National Health Expenditures, National Physician Database, Discharge Abstract Database, Health Insurance Access Database	124,188	C	Statistical imputation (P), probabilistic linkage (S), and categorical matching methods (S)	NR	Comparison with real health data sources by socio-demographic factors including sex, age group, income category, chronic disease status and disability status	NR	No

LSTM: Long Short-Term Memory; GAN: Generative Adversarial Network; CTGAN: Conditional Tabular GAN, WGAN: Wasserstein GAN; WGAN-GP: Wasserstein GAN with Gradient Penalty; FCS: Fully Conditional Specification; CART: Classification and Regression Tree; OSIM2 and ModOSIM: Observational Medical Dataset Simulator II (OSIM2) and its modified version (ModOSIM); C: Cross-sectional; L: Longitudinal; T: Time-series; G: Generative; P: Prediction; S: Sampling; AUROC: Area Under the Receiver Operating Characteristic Curve; AUPRC: Area Under the Precision-Recall Curve; EEG: Electroencephalogram; SVR: Support Vector Regression; RFR: Random Forest Regression; MAE: Mean Absolute Error; RMSE: Root Mean Squared Error; NR: Not reported in the article; The Privacy Assessment column includes privacy risk evaluation methods

### Study purpose

Applications of SD varied. One article examined the ability of SD to replicate results from real data analyses, including statistical estimates and confidence intervals (El Emam et al., 2024). Another article aimed to mitigate covariate bias, defined as imbalance in covariate distributions across groups that can distort study results, to improve the validity of research findings (Juwara et al., 2024). The generation of synthetic administrative health data was also emphasized in two articles. One article developed and applied a method to generate synthetic longitudinal health data (Mosquera et al., 2023). The other article applied two methods to generate SD from administrative sources (Ayilara et al., 2023). Additionally, one article compared SD generation with federated analysis methods to facilitate multi-jurisdictional studies without sharing sensitive raw data (Azizi et al., 2023). In their two-site, multi-jurisdictional study, SD generation took about one month, whereas federated analysis setup took over 1.5 years. The authors highlighted SD as a more efficient method for multi-jurisdictional studies while addressing privacy concerns.

Validating privacy risk metrics was another important application, ensuring that SD do not compromise the confidentiality in real datasets (El Emam et al., 2022). One article evaluated the utility of SD by comparing analysis results for real and synthetic datasets (El Emam et al., 2021). In another article (El Emam et al., 2020a), a methodology was developed and applied to assess the identity disclosure risks associated with fully SD. This model can evaluate the privacy of future SD. Moreover, one source from the grey literature (CanPath, 2021) utilized SD for educational and training purposes, providing hands-on data analysis experiences for students and faculty. In another article (Sallier, 2020), the authors delivered SD with high analytical value for users while maintaining confidentiality. Similarly, another article (Cook et al., 2024) used synthetic electroencephalogram (EEG) data augmentation to expand real datasets with synthetic sample and improve the performance of brain age prediction models.

### Sources of real-world data

Survey data were the most common real-world data source used to generate SD. Specifically, the Canadian Community Health Survey (CCHS), a cross-sectional survey collecting information on health status and healthcare utilization across Canada, was the most common type of health data that was synthesized.

At the provincial level, articles utilized real-world data from Ontario, Alberta, Manitoba, and British Columbia. One study (Mosquera et al., 2023) used linked administrative data from Alberta, drawing from multiple provincial databases including the Provincial Registry and Vital Statistics, Pharmaceutical Information Network, Ambulatory Care Classification System, Discharge Abstract Database, and provincial laboratory data. These datasets were linked using encrypted personal health numbers. Another article (Ayilara et al., 2023) generated SD from Manitoba administrative data, including Manitoba Health Insurance Registry, Hospital Discharge Abstracts Database, Medical Claims Database, and Drug Program Information Network Database. An EEG dataset collected over six years from clinical records across seven hospitals in British Columbia was utilized, demonstrating the integration of clinical data sources into synthetic dataset generation.

In terms of national data, the CCHS was frequently synthesized (Azizi et al., 2023; El Emam et al., 2022, 2024; Hennessy et al., 2015; Juwara et al., 2024). Other national datasets included the Canadian Health Measures Survey (CHMS), the Survey of Household Spending (SHS), the National Health Expenditures (NHEX), and the National Physician Database (NPD), which provided a broad view of population health, healthcare utilization, and expenditures.

Administrative and registry data also contributed significantly to SD generation. The Discharge Abstract Database and the Health Insurance Access Database provided insights into hospitalizations and healthcare access. Additionally, the Canadian Cancer Registry and the Canadian Mortality Registry were leveraged as administrative and surveillance datasets for analyzing cancer incidence, mortality trends, and health outcomes.

### Study sample size

Sample sizes of real data ranged from 903 to 1.6 million. Articles that synthesized administrative health data had larger sample sizes (120,000–1.6 million) compared to those using clinical, survey or public health data, likely reflecting the scale of real-world administrative health data. Privacy risk validation studies use subsets of varying sizes (5,000 to 25,000) to test privacy protection under different conditions. Two articles do not specify a sample size (CanPath, 2021; Sallier, 2020).

### Models and software packages

All articles used fully SD, defined as data entirely generated from models trained on real datasets, with no original records retained. The data synthesis methods fell into three primary categories: generative models, sampling methods, and predictive models. Generative models employ machine-learning techniques to learn and replicate the complex distributions of real-world data. For instance, four articles (Cook et al., 2024; El Emam et al., 2022, 2024; Mosquera et al., 2023) utilized Conditional Tabular Generative Adversarial Networks (CTGANs) and Long Short-Term Memory (LSTM) networks, where CTGANs generate SD by capturing inter-variable relationships, while LSTMs, a type of recurrent neural network, are effective in modeling sequential dependencies. Sampling methods create synthetic datasets by drawing samples from probability distributions derived from real data. One article (Ayilara et al., 2023) employed ModOSIM: Observational Medical Dataset Simulator II (OSIM2) and its modified version (ModOSIM), which simulate healthcare datasets while preserving statistical properties. Synthesized data with ModOSIM closely matched the real data compared to OSIM2. The authors concluded that the ModOSIM can generate synthetic administrative health data consistent with real-world settings. Predictive models generate SD using imputation-based techniques, where missing or synthetic values are inferred based on real data relationships. Nine articles (Azizi et al., 2023; CanPath, 2021; El Emam et al., 2020, 2021, 2022, 2024; Hennessy et al., 2015; Juwara et al., 2024; Sallier, 2020) utilized sequential decision trees, gradient-boosted classification trees, and the ‘synthpop’ R package, which implements classification and regression trees (CART) and fully conditional specification (FCS) for generating realistic SD.

Among these methods, predictive models, particularly sequential decision trees, were the most frequently used. Most articles relied on open-source programming environments, such as R (often using ‘synthpop’) and Python (leveraging CTGAN and LSTM frameworks). One article (Ayilara et al., 2023) mentioned SAS software for implementing OSIM2/ModOSIM. Three articles (El Emam et al., 2024; Juwara et al., 2024; Mosquera et al., 2023) shared illustrative analysis code or provided access upon request; others described their methods without releasing implementation details.

### Evaluation metrics—Utility and privacy risk assessments

Replicability of statistical analyses (e.g., overlapping confidence intervals, effect estimates) was a common way to gauge how well SD reproduced real-data inferences.

One article (El Emam et al., 2024) showed that combining at least ten synthetic datasets—rather than only one— significantly improved the consistency of statistical estimates and analytical decisions, reduced bias, increased confidence interval overlap with the original data, and enhanced statistical power. The article also showed that amplification—repeating the dataset multiple times—offers only marginal additional benefits. Beyond replicability, one article (Hennessy et al., 2015) assessed distribution similarity using socio-demographic variables such as sex, age group, income category, chronic disease status, and disability status. The authors created a representative synthetic dataset, but some limitations existed in data coverage and representativeness. Statistical tests, including the Kolmogorov–Smirnov test, were employed to evaluate the realism of SD compared to real-world distributions (Cook et al., 2024). Three articles (Ayilara et al., 2023; Mosquera et al., 2023; Sallier, 2020) reported that SD preserved analytical value. One article (Sallier, 2020) also noted that SD closely mimicked real data distributions.

To assess how closely the SD matched real-world data, several articles used summary statistics, frequency distributions, concordance coefficients (e.g., Lin’s concordance correlation coefficient), which measure how well two sets of values agree by capturing both precision and accuracy, and distance metrics. Distance metrics, such as the Hellinger distance, quantify the difference between two probability distributions, offering a numerical measure of similarity between synthetic and real data distributions.

Predictive performance metrics, such as Area under the Receiver Operating Characteristic Curve (AUROC), and Area Under the Precision-Recall Curve (AUPRC), were used to evaluate whether models trained on SD performed similarly to models trained on real data (El Emam et al., 2021; Juwara et al., 2024). One article (Cook et al., 2024) evaluated the impact of augmenting real data with SD by measuring predictive performance using metrics such as Mean Absolute Error (MAE) and Root Mean Squared Error (RMSE) across varying proportions of SD used in the model. The authors showed that SD improved model performance. Another article (El Emam et al., 2021) reported only a small decrease in performance when using SD instead of real data.

Several articles assessed different types of privacy risks associated with SD, including membership disclosure risk (reliably determining if a target individual was in the real dataset used for synthesis), attribute disclosure risk (revealing sensitive attributes), and identity disclosure risk (uniquely re-identifying an individual) (Azizi et al., 2023; El Emam et al., 2020, 2021, 2022, 2024; Mosquera et al., 2023). One article (El Emam et al., 2020a) demonstrated that data synthesis significantly reduced identity disclosure risk. Membership disclosure risk was reported to be low across multiple datasets and models (Azizi et al., 2023; El Emam et al., 2021, 2024). Attribute disclosure risk was also reported to be low (El Emam et al., 2021; Mosquera et al., 2023). One article (El Emam et al., 2022) emphasized that, in membership disclosure tests, the proportion of training records used in the attack model—known as the sampling fraction—must match the sampling fraction of the real dataset. Misalignment between these proportions can lead to distorted F1 scores —a measure that balances precision and recall—potentially overestimating or underestimating privacy risk. This can result in misleading conclusions about how much an attacker could realistically learn from the data.

## Discussion

This scoping review aimed to examine how SD have been generated and used in Canadian health research, with a focus on the types of health data synthesized, research purposes, and the methods applied. We identified multiple articles that created SD from Canadian administrative, survey, clinical, and public health datasets.

SD use in Canadian health research is still limited but growing, with most studies focusing on methods related to replicability, bias reduction, and privacy risk mitigation. Health survey data was the most frequently synthesized data type. Provincial administrative and clinical datasets were also used from Alberta, Manitoba, British Columbia, and Ontario. The studies applied a range of synthesis methods, including generative models, sampling techniques, and predictive algorithms. Quality evaluations primarily focused on data utility (e.g., predictive performance), replicability, and disclosure risk.

SD can be implemented faster than federated analysis (Azizi et al., 2023), offering flexibility for exploratory or multi-jurisdictional work. Federated analysis requires major infrastructure, while Trusted Research Environments (TREs) provide the most accurate access under strict oversight. Thus, SD is suited to rapid analyses, federated analysis to cross-site needs, and TREs to maximum-accuracy requirements.

Several articles (Ayilara et al., 2023; El Emam et al., 2021, 2024; Mosquera et al., 2023) included in the review highlight the ability of SD to replicate core statistical properties of real datasets, including overlapping confidence intervals, similar effect sizes, and consistent predictive or inferential models. These findings underscore SD’s potential to preserve the analytical value of real datasets.

The quality and usefulness of SD can vary depending on the method used to generate it and the type of health data being synthesized. Cross-sectional and longitudinal data present distinct challenges for SD generation.

For cross-sectional data, both predictive models (e.g., sequential decision trees) and generative approaches (e.g., CTGANs) were explored. While one article (El Emam et al., 2024) demonstrated that sequential decision trees have better performance than CTGANs, hyperparameter tuning limitations and small sample sizes negatively affect Generative Adversarial Networks (GAN)-based methods. This limitation of generative models such as CTGAN—that they require large datasets to achieve high performance and avoid overfitting—has been noted previously (Afonja et al., 2023). While some models, such as CTGAN, may underperform on smaller datasets, others—like Wasserstein GAN-based architectures—have shown more robust results in specific contexts, such as EEG data (Cook et al., 2024). These differences highlight the importance of selecting generative approaches that align with the characteristics of the target dataset.

For longitudinal data, some articles used LSTM-based methods for synthetic longitudinal data generation (Mosquera et al., 2023). However, existing approaches often fall short in accurately capturing temporal patterns (Mosquera et al., 2023).

Multiple articles confirm that SD can significantly reduce membership and identity disclosure risks (El Emam et al., 2020a, 2021). However, the effectiveness of privacy assessments depends on assumptions about what an adversary might already know about the dataset; if these assumptions are incorrect, the evaluation may not accurately reflect the true privacy risks associated with sharing SD (El Emam et al., 2022). For instance, mismatching the real dataset’s sampling fraction can lead to over- or under-estimation of disclosure risk, potentially resulting in misleading conclusions about privacy protections offered by SD (El Emam et al., 2022).

Beyond membership disclosure, attribute disclosure risks remain a major concern. Low membership disclosure risk does not guarantee low attribute disclosure risk (El Emam et al., 2022), as attackers may still infer sensitive attributes from patterns in the SD. None of the included articles applied differential privacy (DP) or formal privacy accounting; instead, they relied on post-hoc disclosure-risk tests. While DP provides formal privacy guarantees, strict settings can reduce utility. SD generation therefore serves as a complementary approach, and emerging methods are beginning to incorporate DP into SD generators to offer formal guarantees.

There is a fundamental tradeoff in SD generation to balance privacy protection while maintaining statistical accuracy (Bhanot et al., 2021). Strict privacy mechanisms (such as adding more noise or reducing granularity) reduce the likelihood of disclosure, but can diminish utility, while prioritizing accuracy may preserve too much real-world structure and increase re-identification risk. This balance is especially challenging for small or highly-specific populations, including rare disease cohorts, where safeguarding privacy while retaining meaningful patterns is essential. Although SD can reduce reliance on controls like small-cell suppression, risks persist when generators overfit or reproduce distinctive patterns. One approach identified in this review, Synthetic Minority Augmentation (SMA; Juwara et al., 2024), aims to mitigate bias in machine learning models by generating synthetic minority-group data, effectively balancing datasets. It performed best in low-moderate bias settings (≤ 50% missing data) but showed no clear advantage in high-bias scenarios. Techniques like SMA can help mitigate bias, but transparent reporting of real data collection methods, sample size, and inherent limitations is essential for identifying potential bias amplification. Documentation of synthesis methods, including hyperparameters, model settings, and evaluation metrics, is necessary for reproducibility, yet many articles did not provide these details.

### Methodological gaps

Several methodological gaps were evident in the articles. The field remains fragmented, with heterogeneous synthesis methods, validation metrics, and reporting practices, and no consensus on which utility metrics best suit different research purposes (Kaabachi et al., 2025). Reproducibility is limited by incomplete reporting of hyperparameters, model specifications, and data-preparation steps. Bias may also be introduced through reliance on small source datasets. For example, a few included studies used limited sample sizes to generate SD with GANs; however, GANs typically require large training datasets, and insufficient sample size can increase overfitting and reduce the generalizability of the resulting SD. Advanced sequence-based and survival-aware generative models remain underused in Canadian health data, demonstrating an opportunity to adopt longitudinal synthesis methods. Performance variability across different GAN architectures remains a challenge (Yan et al., 2022). Balancing privacy and utility remain unresolved, especially for small subpopulations.

### Recommendations

This review highlights the potential of SD to improve equitable data access and support timely public and population health research in Canada. Although the field continues to emerge, our findings suggest that SD can enable broader use of high-value health datasets while protecting privacy, particularly where interjurisdictional data sharing is restricted. This is especially relevant for multi-jurisdictional studies, health equity surveillance, and policy evaluation. Realizing this potential will require greater methodological consistency and wider application across clinical and public health datasets, ultimately strengthening evidence-informed policy and helping reduce health inequalities in Canada.

### Strengths and limitations

This scoping review has some limitations. Article selection and data extraction were conducted by a single reviewer, which may introduce selection bias, though consultation with a second reviewer helped mitigate this risk. The search was limited to English-language articles, potentially excluding French-language studies. Grey literature was identified through a Google Search, an approach that is inherently non-exhaustive and less reproducible than other approaches. Considerable variability in reporting synthesis methods and privacy metrics also limited cross-study comparability. Despite these limitations, this review has important strengths: it is, to our knowledge, the first to systematically examine SD generated from Canadian health sources, uses a comprehensive search strategy across multiple databases and grey literature, and captures diverse health data types to provide a broad overview of current practices.

## Conclusion

Synthetic health data offer a promising approach to protect privacy and enable secure use of Canadian health data across jurisdictions. Current applications are concentrated in select provincial datasets and national health surveys, and best practices are still emerging. As methods advance, SD may support more secure, scalable, and equitable access to high-value health information for public and population health research in Canada.

## Supplementary Information

Below is the link to the electronic supplementary material.

ESM 1 (PDF 30.4 KB)

ESM 2 (DOCX 83.9 KB)

## Data Availability

Not applicable.
