# Supplementary material for: Synthetic health data in Canada: A scoping review of methods, applications, and data sources
Source: Can J Public Health. 2026 Feb 9;117(5):996–1009. doi: 10.17269/s41997-026-01153-6 (PMC13680348; doi:10.17269/s41997-026-01153-6)
Supplement: Supplementary file 1 [file 41997_2026_1153_MOESM1_ESM.pdf]

Synthetic Health Data in Canada: A Scoping Review of Methods, Applications, and Data Sources

Journal Name: Canadian Journal of Public Health

Online Resource 1: Detailed search expression used for databases (up to September 2024)

For PubMed:

("fake"[Title] OR "synthetic"[Title] OR "simulation"[Title] OR "simulated"[Title] OR "synthesis"[Title] OR "synthesizing"[Title] OR "synthesizer"[Title] OR "generating"[Title] OR "generative"[Title] OR "privacy"[Title]) AND (("synthetic"[Title/Abstract] OR "synthesis"[Title/Abstract] OR "synthesizing"[Title/Abstract] OR "synthesizer"[Title/Abstract] OR "generating"[Title/Abstract] OR "generative"[Title/Abstract]) AND ("administrative health data"[Title/Abstract] OR "administrative record"[Title/Abstract] OR "administrative records"[Title/Abstract] OR "administrative dataset"[Title/Abstract] OR "administrative datasets"[Title/Abstract] OR "administrative data"[Title/Abstract] OR "administrative claim"[Title/Abstract] OR "administrative claims"[Title/Abstract] OR "health data"[Title/Abstract] OR "health record"[Title/Abstract] OR "health records"[Title/Abstract] OR "healthcare data"[Title/Abstract] OR "longitudinal health data"[Title/Abstract] OR "healthcare database"[Title/Abstract] OR "healthcare databases"[Title/Abstract] OR "healthcare claim"[Title/Abstract] OR "healthcare claims"[Title/Abstract] OR "administrative health data"[Title/Abstract] OR "clinical data"[Title/Abstract] OR "survey data"[Title/Abstract])) AND (Canada[Affiliation] OR Canadian[Affiliation])

For Scopus:

(TITLE(fake OR synthetic OR simulation OR simulated OR synthesis OR synthesizing OR synthesizer OR generating OR generative OR privacy)) AND ((TITLE-ABS(synthetic OR synthesis OR synthesizing OR synthesizer OR generating OR generative)) AND TITLE-ABS("administrative health data" OR "administrative record" OR "administrative records" OR "administrative dataset" OR "administrative datasets" OR "administrative data" OR "administrative claim" OR "administrative claims" OR "health data" OR "health record" OR "health records" OR "healthcare data" OR "longitudinal health data" OR "healthcare database" OR "healthcare databases" OR "healthcare claim" OR "healthcare claims" OR "clinical data" OR "survey data")) AND (AFFIL(Canada) OR AFFIL(Canadian))

For Web of Science:

TI=(fake OR synthetic OR simulation OR simulated OR synthesis OR synthesizing OR synthesizer OR generating OR generative OR privacy) AND TS=(synthetic OR synthesis OR synthesizing OR synthesizer OR generating OR generative) AND TS=("administrative health data" OR "administrative record" OR "administrative records" OR "administrative dataset" OR "administrative datasets" OR "administrative data" OR "administrative claim" OR "administrative claims" OR "health data" OR "health record" OR "health records" OR "healthcare data" OR "longitudinal health data" OR "healthcare database" OR "healthcare databases" OR "healthcare claim" OR "healthcare claims" OR "clinical data" OR "survey data") AND (AD=Canada OR AD=Canadian)

For Google:

("synthetic data" OR "data synthesis" OR "simulated data" OR "generative data" OR "data privacy") AND ("administrative health data" OR "healthcare data" OR "clinical data" OR "survey data") AND (Canada OR Canadian)
